# Impact of covid on riots and associated behaviors in the united states

**DOI:** 10.1192/j.eurpsy.2021.801

**Published:** 2021-08-13

**Authors:** R. Mueller

**Affiliations:** Forensic Psychiatry, Community Health Network, Greenfield, United States of America

**Keywords:** COVID associated Criminal Conduct, Riots, forensic psychiatry, Antisocial Behavior

## Abstract

**Introduction:**

The racial riots of 2020 in the US, beginning in Minneapolis, had a global impact inciting protests internationally. We look at the impact of COVID, the social isolation and frustration that therefore existed and how this effected the instigation of the riots.

**Objectives:**

--To review the history of racism in the United States and the abolition theories, comparing US and UK. --To consider the impact of international immigration on the cultural tension in the US; Minnesota accepted a large population of Somalis in 1992 as refugees. --To explore how this progress toward racial equality has stagnated under the leadership of President Donald Trump. --To look at how COVID in the context of the above historical factors has served as a unwitting catalyst to racial riots and global protests.

**Methods:**

Literature research including historical accounts of principles of abolition, post-civil war reconstructive political manuevers, 1950’s segregation protests and political supports (US and UK), refugee relief efforts made by the US [specifically related to Somalia], and reports regarding the impact of COVID on the 2020 reaction to racial injustice.

**Results:**

Evidence suggests that across time periods, recourses of politicians [US and global] resulted in negative relations internationally with respect to immigration. The unique situation created by COVID resulted in a crucible effect following the death of George Floyd.

**Conclusions:**

Previous attempts at creating equality have proven unsuccessful and apathetic on the part of those in power. This has lead to a situation where COVID created a perfect storm in order to ignite racial tensions in the US.

